# Comparative pharmacokinetics and safety assessment of transdermal berberine and dihydroberberine

**DOI:** 10.1371/journal.pone.0194979

**Published:** 2018-03-26

**Authors:** Beth Buchanan, Qingfang Meng, Mathieu-Marc Poulin, Jonathan Zuccolo, Chike Godwin Azike, Joseph Gabriele, David Charles Baranowski

**Affiliations:** Research and Development, Delivra Corp., Charlottetown, Prince Edward Island, Canada; Universidade do Porto, Faculdade de Farmácia, PORTUGAL

## Abstract

The natural alkaloid berberine has been ascribed numerous health benefits including lipid and cholesterol reduction and improved insulin sensitivity in diabetics. However, oral (PO) administration of berberine is hindered by poor bioavailability and increasing dose often elicits gastro-intestinal side effects. To overcome the caveats associated with oral berberine, we developed transdermal (TD) formulations of berberine (BBR) and the berberine precursor dihydroberberine (DHB). These formulations were compared to oral BBR using pharmacokinetics, metabolism, and general safety studies *in vivo*. To complete this work, a sensitive quantitative LC-MS/MS method was developed and validated according the FDA guidelines for bioanalytical methods to simultaneously measure berberine, simvastatin, and simvastatin hydroxy acid with relative quantification used for the berberine metabolite demethylene berberine glucuronide (DBG). Acute pharmacokinetics in Sprague-Dawley rats demonstrated a statistically relevant ranking for berberine bioavailability based upon AUC_0-8_ as DHB TD > BBR TD >> BBR PO with similar ranking for the metabolite DBG, indicating that transdermal administration achieves BBR levels well above oral administration. Similarly, chronic administration (14 days) resulted in significantly higher levels of circulating BBR and DBG in DHB TD treated animals. Chronically treated rats were given a single dose of simvastatin with no observed change in the drugs bioavailability compared with control, suggesting the increased presence of BBR had no effect on simvastatin metabolism. This observation was further supported by consistent CYP3A4 expression across all treatment groups. Moreover, no changes in kidney and liver biomarkers, including alanine aminotransferase and alkaline phosphatase, were observed between treatment formats, and confirming previous reports that BBR has no effect on HMG-CoA expression. This study supports the safe use of transdermal compositions that improve on the poor bioavailability of oral berberine and have the potential to be more efficacious in the treatment of dyslipidemia or hypercholesterolemia.

## Introduction

Berberine (BBR) is a natural alkaloid found in a variety of plant species including barberry (*Berberis*), meadow rue (*Thalictrum*), celandine (*Chelidonium*), and goldenseal (*Hydrastis canadensis*) [1–4]. BBR has been investigated for a variety of health benefits including anti-microbial [5,6], anti-inflammatory [7], chemotherapeutic [8], anti-diabetic [9,10], as well as cholesterol and lipid-lowering properties [11,12]. Regarding the latter, berberine is widely used in humans as a natural supplement to aid the management of dyslipidemia [13]. The anti-microbial activity has a pronounced effect on the gut microbiota and often translates into adverse events or dose reduction in clinical trials investigating the value of BBR treatment for diabetes and dyslipidemia [10,14,15]. The need to reduce dosage in these studies relates to the high levels of BBR required to achieve effectiveness (1,000–1,500 mg per day), a result of the poor bioavailability of oral BBR [16,17].

Several oral formulation techniques such as encapsulation [18,19], spray drying [20] and the co-administration with P-glycoprotein inhibitors [9], have been used to attempt to overcome the inherently poor bioavailability of BBR. For example, Yu et. al., demonstrated a polyethylene glycol and phosphatidylcholine encapsulation of oral BBR with improved pharmacokinetic parameters including the peak serum concentration (C_max)_ and area under the curve (AUC) by ~3-fold [18]. However, oral formulations may unavoidably encounter gastrointestinal side effects and the use of complex non-medicinal excipients or processes may complicate the clinical translation and commercialization of such products.

In 2008, Kong et al showed that oral BBR had lipid lowering effects when used in combination with statin drugs in hyperlipidemic rats [21]. This precedent led to the hypothesis that berberine could be used alone or in conjunction with statins for the treatment of dyslipidemia. Additionally, preclinical studies have suggested BBR may be hepatoprotective from a variety of chemical insults [22–24]. For example, Germoush and Mahmoud demonstrated that BBR rescued the hepatic damage associated with cyclophosphamide treatment [24]. Indeed, BBR has been shown to affect the metabolism of drugs through changes in CYP3A4 expression [25]. The effects of BBR on CYP3A4-mediated drug metabolism is of particular interest as this enzyme degrades several widely used statin drugs (e.g. simvastatin and lovastatin) that may be concomitantly used with BBR to treat dyslipidemia or hypercholesterolemia [25,26]. Although an enhanced BBR formulation may yield an increase in bioavailability, it may also generate unwanted side-effects such as alterations in hepatic health and metabolism of other compounds.

To overcome concerns of bioavailability, gastrointestinal side effects, and investigate the safety and drug interactions of BBR we developed transdermal formulations of BBR and the reduced derivative, dihydroberberine (DHB). Simultaneous pharmacokinetic analysis of BBR and simvastatin using liquid chromatography-tandem mass spectrometry (LC-MS/MS) allowed us to compare bioavailability via different delivery routes as well as to investigate any possible interactions between BBR or DHB with this statin drug.

## Experimental

The analytical method for the quantification of berberine, simvastatin and simvastatin hydroxy acid described within was developed and validated according to the FDA bioanalytical method validation guidance document [27]. No analytical standard for demethylene berberine glucuronide (DBG) was available, and therefore the quantification method for that metabolite was not validated.

### Chemicals and reagents

Berberine chloride hydrate (14050), potassium carbonate (P5833), sodium hydroxide (S8045) and chloroform (650498) were purchased from Sigma Aldrich. Pharmaceutical compounding grade isopropyl myristate (8170662500) and polysorbate-20 (8170721000) were purchased from EMD-Millipore. Simvastatin (SIM, S485000), simvastatin hydroxy acid (SHA, S485020), d_6_-simvastatin (d6-SIM, S485002), d_6_-simvastatin hydroxy acid (d6-SHA, S485022), and d_6_-berberine hydrochloride (d6-BBR, B318152) were purchased from Toronto Research Chemicals. Methanol (MX0488-1), water (Millipore-HPLC grade), and sodium borohydride (71321) were purchased from VWR. The transdermal base cream DelivraSR (13750–2) is manufactured by Delivra Corp. and is available commercially for compounding purposes.

### Instrumentation

An ABSciex 5500 Qtrap mass spectrometer with a Turbo V source equipped with an Agilent 1260 HPLC system was used for the analysis with Analyst 1.6.2 software.

### Chromatographic conditions

Chromatic separation was performed with a C18 HPLC column (Zorbax eclipse XDB—4.6x150 mm, 5 μm) with a mobile phase gradient consisting of 0.4% (v/v) formic acid in water (phase A) and 0.2% (v/v) formic acid in methanol (phase B), at a flow rate of 0.75 mL/min. The first 6 minutes of the gradient program was isocratic with 45% A and then phase B was increased to 100% by 9 minutes and held at 100% until 13 minutes. The initial conditions (45% A) were then re-equilibrated for 5 minutes. The sample injection volume was 10 μL, the column temperature was 40 °C and the autosampler temperature was maintained at 20 °C.

### Mass spectrometer conditions

The mass spectrometer was operated in bipolar mode, observing analytes BBR, d6-BBR, DBG, SIM, d6-SIM, SHA and d6-SHA using the parameters outlined in Tables 1 and 2. A multiple reaction monitoring (MRM) pair for DHB was not included in the analysis because it is quickly oxidized to BBR once absorbed through the skin and into the bloodstream [28].

**Table 1:** Retention time and MRM pairs for analytes.

Identity	Q1 Mass (Da)	Q3 Mass (Da)	Time (msec)	Mode	Retention time (min)
BBR	336.08	292.1	100	+	2.76
D6-BBR	342.2	306.05	100	+	2.76
DBG	500.1	324.1	100	+	2.16
SIM	419.27	199.1	100	+	12.77
D6-SIM	425.27	199.1	100	+	12.77
SHA	435.3	319.1	100	_	12.61
D6-SHA	441.3	319.1	100	_	12.61

**Table 2:** LC-MS/MS parameter table.

Parameters	1^st^ period	2^nd^ period positive mode	2^nd^ period negative mode
Time (min)	0–9	9–18	9–18
DP	110	40	-40
EP	10	10	-12
CE	45	17	-22
CXP	10	15	-10
CUR	10	10	10
CAD	Medium	Medium	Medium
IS	5500	5500	-4500
TEM	600	400	400
GS1	50	50	50
GS2	50	50	50
Resolution	Low	Unit	Unit

### Preparation of standard solutions

Stock solutions of SHA, d6-SHA and were prepared by dissolving standards with authenticated mass of 1 mg in 1 mL of 50:50 methanol:water to generate a solution of 1 mg/mL. Stock solutions of d6-BBR, SIM and d6-SIM were prepared by dissolving standards with authenticated mass of 1 mg in 2 mL of 25:25:50 methanol:water:acetonitrile to generate a solution of 0.5 mg/mL. A stock solution of BBR was prepared by dissolving 5 mg in 5 mL of 25:25:50 methanol:water:acetonitrile to generate a solution of 0.5 mg/mL.

### Calibration curves and quality control samples

The samples for standard calibration curves were prepared by spiking blank rat serum (95 μL) with 5 μL of an appropriate concentration of working solution to yield serum concentrations of 0.49, 0.98, 1.95, 3.91, 7.81, 15.63, 31.25, 62.5, 125, 250 and 500 ng/mL of berberine, simvastatin and simvastatin hydroxy acid. Quality control samples were prepared from blank serum at concentrations of 1.4, 5, 25 and 125 ng/mL.

### Serum sample preparation

Serum samples, calibration standards and QC samples were processed using a liquid-liquid extraction. The serum samples were removed from -80 °C storage and allowed to thaw at room temperature for 10 minutes. After vortexing, 40 μL of serum was treated with 200 μL solution of acetonitrile containing 3% v/v acetic acid, 6.25 ng/mL of d6-BBR, and 31.25 ng/mL of both d6-SIM and d6-SHA. The samples were then vortexed and centrifuged. The supernatant was evaporated to dryness under vacuum at room temperature. The residue was reconstituted in 80 μL of 50:50 methanol:water assisted by vortexing and centrifugation. Berberine samples with berberine concentrations above the ULOQ were diluted to ¼ in blank serum extract.

### Method validation

Method validation was conducted according to the FDA guidance for bioanalytical method validation [27].

#### Selectivity

Assay selectivity was evaluated by analyzing blank pooled rat serum, as well as serum from 6 animals used in the study prior to treatment.

#### Calibration curves, LLOQ and ULOQ

The lower limit of quantification (LLOQ) for each analyte was determined usingto the US Food and Drug Administration (FDA) bioanalytical guidelines using the following criteria: the mean concentration of the analytes was within 15% of the nominal concentration at LLOQ, the precision of quality control (QC) samples did not exceed 15% coefficient of variation (CV), and peak area of the analytes at the LLOQ was > 5 times the blank peak area for each analyte. The upper limit of quantification (ULOQ) required that the mean calculated concentration of the analytes was within 15% of the nominal concentration.

#### Assay precision and accuracy

Precision and accuracy of the assay were determined by replicate analysis (n = 6) of quality control samples on the same day (intra-day) and on three separate days (inter-day). Precision (CV %) was calculated from standard deviation (SD) and mean observed concentration (C_obs_) with the calculation CV % = (SD/C_obs_) x 100%. The accuracy was calculated from nominal concentration (C_nom_) and mean observed concentration with the calculation bias (%) = [(C_obs_-C_nom_)/ (C_nom_)] x 100%.

#### Extraction recovery and matrix effect

Extraction recovery of analytes from rat serum (N = 6) was assessed by comparing the peak area of the extracted analyte to those of blank serum extracts spiked with standard solutions. The extraction recoveries of QC samples at three concentrations were calculated as the percent of peak area of extracted analyte to peak area of spiked analyte. Matrix effects on analytes were assessed by comparing the peak area of analytes in the extraction solvent with peak areas of analytes of the same concentration in blank serum extracts (N = 6).

#### Stability

The stability of BBR, SIM, and SHA was evaluated under conditions expected to be encountered during storage, transportation, processing and analysis of samples. Six replicates of QC samples (spiked serum) were analyzed for each analyte at each concentration after being subjected to the following sets of conditions: three freeze/thaw cycles at—20 °C, long term freezing storage (4 weeks at—80 °C), and 3 hours at room temperature. Stock solutions and samples prepared in serum extracts were also monitored for stability for up to 5 days at room temperature.

### Dihydroberberine synthesis and formulation construction

DHB was synthesized according to a published method with slight modifications [29]. Briefly, a sodium borohydride (0.5 g) solution in 5% aqueous sodium hydroxide was added dropwise to a stirring solution of berberine chloride hydrate (5.0 g) and potassium carbonate (6.5 g) in methanol (100 mL) under nitrogen. The reaction was stirred for one hour at room temperature. The precipitate was collected using suction filtration and washed with methanol (30 mL). The precipitate was then dissolved in chloroform (20 mL), immediately filtered and the solvent removed *in vacuo*. The crude product was crystallized from ethanol under nitrogen and stored in a desiccator. Each lot was evaluated for purity using ^1^H NMR, with a minimum of 90% purity required for use in formulation. Select lots were evaluated for boron content using elemental analysis.

Topical formulations of DHB and BBR (5% (w/w)) were prepared under a continuous flow of nitrogen. DHB or BBR (2.0 g) was ground for 5 minutes in a mortar and pestle. Isopropyl myristate (3.576 mL) and polysorbate 20 (172 μL) were added to the mortar and this mixture was macerated for 5 minutes. Delivra SR (36.6 g) was added and the mixture was macerated for an additional 5 minutes This crude formulation was further homogenized by mechanical compression through two tandem 50 mL syringes coupled with a 4.1 mm ID adapter using 20 strokes. The formulation was stored in an airless pump at 4 °C for the duration of the animal study. An equivalent vehicle formulation was prepared in the same manner without the addition of BBR or DHB. Formulations containing BBR or DHB were analyzed for stability over the course of the study (variation in concentration of active <10%).

### Animal studies

The animal protocols for the studies were conducted by a private contract research organization and approved by internal animal care and utilization committee (IACUC) in accordance with the principles of the Animal for Research Act of Ontario and the guidelines of Canadian Council on Animal Care (CCAC). Animals were individually housed with standard bedding and enrichment. Standard chow and water were provided *ad libitum* with the exception of orally dosed animals who were deprived of food overnight and fed approximately 2 hours following dosing. General health was monitored throughout the chronic study with daily body weight measurements, daily health observations, and a Functional Observation Battery (FOB) [30] was conducted on Days -1, 6, and 13 in relation to the course of article administration.

### Acute pharmacokinetic study

Nineteen Male Sprague-Dawley (SD) rats weighing between 250–375 g were provided by Charles River Laboratories. Animals were acclimated to the testing facility for a minimum of 1 week prior to initiation of test article administration. The study was designed with three treatment groups comprised of berberine oral gavage (BBR PO), berberine topical (BBR TD), and dihydroberberine topical (DHB TD) using groups of three, eight, and eight animals for each treatment respectively. The day prior to test article administration, all animals were catheterized at the carotid artery according to standard operating procedures and allowed to recover from surgery. Oral BBR was prepared as an aqueous stock solution of 20 mg/mL BBR with 0.5% (w/v) carboxymethyl-cellulose and animals received a single bolus of 90 mg/kg of the active. For BBR TD and DHB TD a 2x2 inch area caudal to shoulder blades and along the midline was shaved and a mass of 1.8 g/kg (equal to 90 mg/kg active given a 5% (w/w) formula) of the topical was applied uniformly. Blood was collected via the catheter at 9 time-points (t = 0, 0.5, 1, 2, 2.5, 3, 3.5, 4, and 8 hours) into serum tubes and processed for serum according to standard procedures. Serum samples were maintained at -80 °C until analysis.

### Chronic pharmacokinetic study

Forty male Sprague-Dawley rats weighing between 250–375 g were provided by Charles River Laboratories. Animals were acclimated to the testing facility for a minimum of 1 week prior to initiation of test article administration. The five treatment groups included berberine oral gavage (BBR PO), berberine topical (BBR TD), dihydroberberine topical (DHB TD), vehicle topical (vehicle TD), and untreated control (CTRL). Rats were randomly assigned to each treatment condition with eight animals per group. Using the same dosage parameters (oral BBR at 90 mg/kg and all transdermal formulations at 1.8 g/kg) animals received test articles or vehicle TD for 14 consecutive days, once daily in the morning. On day 14 each group received test article and half (N = 4) underwent terminal blood collection (two hours post-administration) via cardiac puncture with ~500 μL processed to yield serum and frozen at -80 °C and the remainder submitted for clinical chemistry testing (Antech Diagnostics). In addition, the liver was removed, flash frozen in liquid nitrogen and maintained at -80 °C until time of analysis. On day 15 the remaining animals (N = 4/group) received test article or vehicle TD, were catheterized at the carotid artery according to standard operating procedures and allowed to recover from surgery. On day 16 animals received test article or vehicle TD (time zero) then dosed orally by gavage with 12 mg/kg of simvastatin one hour later. Blood was collected via the catheter at 10 time-points (t = 0, 1, 1.5, 2, 3, 3.5, 4, 4.5, 5, and 9 hours) into serum tubes and processed for serum according to standard procedures. Serum samples were maintained at -80 °C until analysis.

### Pharmacokinetic data analysis

Microsoft Excel 2013, with a non-compartment model was used to calculate all the pharmacokinetic parameters. The concentration of SIM and SHA in last 6-time points for each treatment group were converted to the natural logarithm. The slope of the semilogarithmic line of ln(concentration) vs. time was assigned as the elimination rate constant (Kel). Half life (T_1/2_) was calculated using the equation: T_1/2_ = - 0.693 / Kel. Area under the curve (AUC_0-8_) was calculated by first, multiplying half of summary of concentration at t_n_ and t_n-1_ by the difference value of the two-time points. Then, AUC_0-8_ was calculated by summing the AUC_tn-(tn-1)_ calculated concentrations from t_0_-t_8_. The maximum concentration (C_max_) was chosen as the highest average serum concentration for each group, and the time at which it was observed was assigned the value t_max_. Statistical analysis was performed using Graphpad Prism 6 software using an unpaired, one-way analysis of variance (ANOVA) in conjunction with Tukey’s range test on log-transformed data. Unpaired t-tests were used to compare equivalent test articles (AUC) between chronic and acute settings.

### Tissue sample preparation and immunoassays

As described, livers from animals having received a test article or vehicle for 14 consecutive days were removed and flash frozen in liquid nitrogen and maintained at -80 °C until analysis. Two livers per treatment group were evaluated for CYP3A4 and HMG-CoA reductase using a combination of enzyme-linked immunosorbent assays (ELISA) and western blot techniques. Liver lysates were made to 25 μL/mg (Dulbeco’s phosphate-buffered saline (DPBS)/tissue mass), then homogenized with tissue homogenizer for 20 seconds on ice. Lysates were sonicated at room temperature for 30 seconds, then centrifuged at 4 °C at 5000 xg for 5 min. Clear supernatant was retrieved, aliquoted, and stored at -20 °C until analysis by ELISA or western blot.

For ELISA quantification of CYP3A4 and HMG-CoA reductase were performed according to manufacturer’s instructions (LifeSpan BioSciences LS-F22383 and LifeSpan BioSciences LS-F15758). Four technical replicates were used for each liver sample. Data was processed using Graphpad Prism 6 software, with a 4-parameter curve fit generating the standard curve used for interpolation. Graphpad Prism 6 software was also used for data analysis and figure generation. Statistical analysis was performed using an unpaired, one-way ANOVA in conjunction with Tukey’s range test.

For western blot analysis, Pierce^™^ BCA protein quantification assay (Thermo 23227) was performed on the rat liver lysates to determine total protein concentration. Lysates were diluted to 1/100 in deionized water and ran in triplicates following manufacturer instructions. Standard series was prepared using deionized water as diluent and ran in duplicate following manufacturer instructions. After 30 minutes of reaction, absorbance at 560 nm was measured. Data was processed using Graphpad Prism 6 software, with a 4-parameter curve fit generating the standard curve used for interpolation. Equal amounts of total protein (40 μg) of each sample was loaded in duplicates into the wells of pre-cast 10% (w/v) polyacrylamide SDS-PAGE gels (biorad 4561034DC) in reducing conditions along with a molecular weight ladder (biorad 161–0374). Electrophoresis was performed at 200 V for 40 minutes using the biorad mini-PROTEAN Tetra Vertical Electrophoresis Cell System. Gels were blotted to polyvinylidene fluoride (PVDF) membrane at 90 V for 90 minutes. Membranes were blocked with blocking buffer (3% bovine serum albumin in TBST (tris-buffered saline (SkyTek TBS999) with 0.1% Polysorbate 20) for 2 hours at RT (room temperature) with shaking. Primary antibody incubation overnight at 4 °C (rabbit-anti-cyp3A4 Novus NB600-1396 at 1/1000 dilution in 15 mL TBST or rabbit-anti-HMG-CoA reductase (Millipore ABS229) at 1/1000 dilution in 15 mL TBST. Wash with 3x5 minutes TBST, then secondary antibody incubation for 2 hours at RT with shaking (Goat-anti-Rabbit-HRP Cayman 10004301 at 1/1000 in 15 mL TBST). Wash with 3x5 minutes TBST, then ECL imagining with UVP VisionWorks imaging station (clear filter, no transillumination), using 2 mL of Pierce^™^ chemiluminescent substrate (Thermo 34077) with an exposure time of 10 minutes. Membranes were covered in stripping buffer (Deionized water with 1.5% (p/v) Glycine, 1% (v/v) polysorbate 20, 0.1% (p/v) SDS, pH adjusted to 2.2 using HCl) 2x10 minutes with shaking at RT, washed with DPBS for 2x10 minutes at RT with shaking, then washed with 2x5 minutes TBST at RT with shaking. Membranes were blocked with blocking buffer overnight at 4 °C. Primary antibody incubation for 2 hours at RT with shaking (Mouse-anti-β-actin Sigma A5441 at 1/2000 in 15 mL TBST). Wash with 3x5 minutes TBST, then secondary antibody incubation (Goat-anti-mouse-HRP Santa Cruz sc-2005) for 2 hours at RT with shaking. Wash with 3x5 minutes TBST, then ECL imagining with UVP VisionWorks imaging station (clear filter, no transillumination), using 2 mL of Pierce^™^ chemiluminescent substrate (Thermo 34077) with an exposure time of 1 minute. Image processing with Image J 1.51r and GIMP 2.8 software.

Western blot quantification using ImageJ 1.51r software was performed by comparing Mean Grey Value of analyte (cyp3A4 or HMG-CoA Reductase) bands (at the expected molecular weight) with constant Region of Interest (ROI) dimensions, using as reference signal the Mean Grey Value of the corresponding β-actin bands. Graphpad Prism 6 software was used for data analysis and figure generation. Statistical analysis was performed using an unpaired one-way ANOVA in conjunction with Tukey’s range test.

## Results and discussion

### Optimization of LCMS conditions for quantitative analysis

The low volume of serum available from the rodents motivated the development of a sensitive method that accommodated the measurement of all three analytes at once. The mobile phase gradient was developed to facilitate the analysis of the two families of compounds, generate good peak shape and resolution, and minimize matrix effects. Bipolar mode was used to optimize ionization for the three analytes. In the positive mode, molecular ions were most abundant for berberine [M]^+^ and simvastatin [M+H]^+^ and their corresponding internal standards. In the negative mode, the molecular ion was the most abundant for simvastatin hydroxy acid [M-H]^-^ and its internals standard. Deuterated internal standards were chosen for the study. A published MRM pair for a known berberine rat liver metabolite: demethylene berberine glucuronide [31] was included in the method to identify if it was being produced and to measure its relative concentration.

Using protein precipitation, 3% (v/v) acetic acid in acetonitrile, was successful at simultaneous extraction of all the analytes and removal of unwanted components from serum.

### Method validation

**Fig 1:**
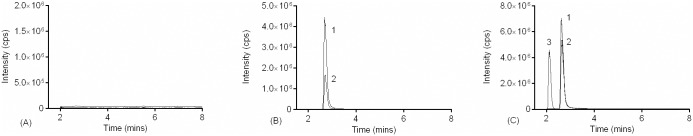
LC-MS/MS profiles of serum samples with BBR introduced *in silico and in vivo*. Chromatograms of (A) blank serum (B) blank serum spiked with (1) d6-BBR (31.25 ng/mL) and (2) BBR (7.8 ng/mL), and (C) serum from a Sprague-Dawley rat treated with 90 mg/kg BBR TD and collected 0.5 hours post administration demonstrating the presence of internal control (1) d6-BBR, (2) BBR, and the glucuronide metabolite (3) DMG.

**Fig 2:**
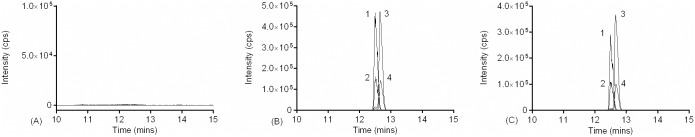
LC-MS/MS profiles of serum samples with SIM and SHA introduced *in silico* and *in vitro*. Chromatograms of (A) blank serum (B) blank serum spiked with (1) d6-SHA (156.25 ng/mL), (2) SHA (62.5 ng/mL), (3) d6-SIM (156.25 ng/mL) and (4) SIM (62.5 ng/mL) and (C) serum samples 2.5 hours after oral administration of SIM (1) d6-SHA (spiked—156.25 ng/mL) (2) SHA (3) d6-SIM (spiked—156.25 ng/mL) and (4) SIM.

The FDA bioanalytical guidance document provided the framework for the validation of the LC/MS/MS method. Blank serum samples were found to be free of interference with the compounds of interest. Representative chromatograms of blank serum extract, BBR, d6-BBR, DBG, SIM, d6-SIM, SHA and d6-SHA are shown in Figs 1 and 2. The retention times of BBR, DBG, SIM and SHA were 2.8, 2.1, 12.6 and 12.8 mins respectively.

The LLOQ was 0.49 ng/mL for BBR, SIM, and SHA and the ULOQ was 62.5 ng/mL for BBR and 500 ng/mL for both SIM and SHA. This is comparable to literature values [32–36] with some LLOQs reported as low as 0.1 ng/mL for BBR, SIM or SHA. The calibration curves for SIM and SHA were constructed using weighted linear regression of the peak area ratio of the analytes to their corresponding internal standard (IS) vs. serum concentration of analytes over the range of 0.49 ng/mL to 500 ng/mL with regression equations: y = 0.0046x + 0.0048 for SIM and y = 0.0071x+0.0059 for SHA. The calibration curve for BBR was constructed using a weighted quadratic curve of the peak area ratio of the analytes to their corresponding IS vs. serum concentration of analytes over the range of 0.49 ng/mL to 62.5 ng/mL. The BBR standard series was assigned the closest possible linear curve from 0.49–62.5 ng/mL to use to calculate a relative concentration to DBG from its ratio of peak area to d6-BBR peak area.

The intraday and interday precision and accuracy results are summarized in Table 3. All values were within recommended limits. Extraction recoveries analyzed at three concentrations for each analyte were greater than 84.6% for all analytes (Table 4). Matrix effects were low for BBR and SIM but more pronounced for SHA. Liu et al. reported a combined method for evaluation of BBR, SIM and SHA with low matrix effects for all analytes in plasma [36]. The current method controlled for the SHA matrix effects using deuterated internal standards.

**Table 3:** Precision and accuracy of berberine, simvastatin and simvastatin hydroxy acid in rat serum (inter-day n = 6x3; intra-day n = 6).

Sample	Nominal Concentration (ng/mL)	Measured Concentration (ng/mL) (mean +/- SEM)	CV (%)	Bias (%)
Berberine				
Interday	1.4	1.33 +/- 0.03	10.1	-4.8
	5	5.04 +/- 0.09	7.5	1.0
	25	25.28 +/- 0.25	4.2	1.1
	125 (with dilution)	127.44 +/- 1.42	4.6	2.0
Intraday	1.4	1.38 +/- 0.01	1.4	-1.4
	5	5.16 +/- 0.04	1.8	3.3
	25	25.30 +/- 0.44	4.3	1.2
	125 (with dilution)	126.13 +/- 0.75	1.4	0.9
Simvastatin				
Interday	1.4	1.35 +/- 0.02	6.5	-3.4
	5	5.44 +/- 0.05	3.5	8.7
	25	27.24 +/- 0.28	4.4	9.0
	125	136.60 +/- 1.01	3.1	9.3
Intraday	1.4	1.41 +/- 0.01	2.0	0.8
	5	5.25 +/- 0.07	3.4	5.0
	25	26.23 +/- 0.59	5.5	4.9
	125	132.50 +/- 0.88	1.6	6.0
Simvastatin hydroxy acid				
Interday	1.4	1.28 +/- 0.02	7.7	-8.5
	5	4.98 +/- 0.12	9.8	-0.4
	25	25.91 +/- 0.37	6.0	3.6
	125	122.05 +/- 1.56	5.4	-2.4
Intraday	1.4	1.40 +/- 0.03	4.6	-0.2
	5	4.92 +/- 0.06	2.7	-1.4
	25	24.67 +/- 0.18	1.8	-1.3
	125	121.83 +/- 1.97	3.9	-2.5

**Table 4:** Extraction recovery and matrix effect of BBR, SIM and SHA (n = 6) and d6-BBR, d6-SIM and d6-SHA (n = 6x3).

Sample	Concentration (ng/mL)	Recovery (%) +/- SEM	Matrix Effect (%) +/- SEM
Berberine	1.4	88.1 +/- 2.3	96.9 +/- 1.5
	5	87.0 +/- 3.9	89.6 +/- 2.8
	25	90.8 +/- 0.9	102.2 +/- 1.2
D6-berberine	31.2	88.2 +/- 2.7	101.0 +/- 2.3
Simvastatin	1.4	99.6 +/ - 2.8	85.3 +/- 7.7
	25	105.8 +/- 1.1	84.5 +/- 2.0
	125	84.6 +/- 6.5	109.2 +/- 8.6
D6-simvastatin	156.25	101.9 +/- 3.2	86.9 +/- 4.0
Simvastatin hydroxy acid	1.4	96.2 +/- 2.9	41.8 +/- 2.8
	25	99.7 +/- 3.0	32.7 +/-0.5
	125	103.6 +/- 6.4	30.3 +/- 1.7
D6-simvastatin hydroxy acid	156.25	93.6 +/- 2.6	31.2 +/- 1.0

The stability of BBR during sample processing has been described previously [37,38], however, SIM has been identified to convert (enzyme and/or non-enzymatic) to SHA [39], introducing a confounding factor. While this reaction can be reduced through additional blood handling protocols we chose to quantify both analytes (SIM and SHA) to cumulatively reflect this drugs pharmacokinetics. Importantly, SIM and SHA were found to be stable in stock solutions, once biological samples began the extraction process, and maintained a concentration within +/- 15% of their initial concentration (S1 Table). Berberine and simvastatin hydroxy acid showed acceptable stability in serum at room temperature, during freeze thaw cycles, in stock solutions and in processed samples with responses that varied no more than 11% of original concentration after subjection to test conditions.

### Acute berberine pharmacokinetics

**Fig 3:**
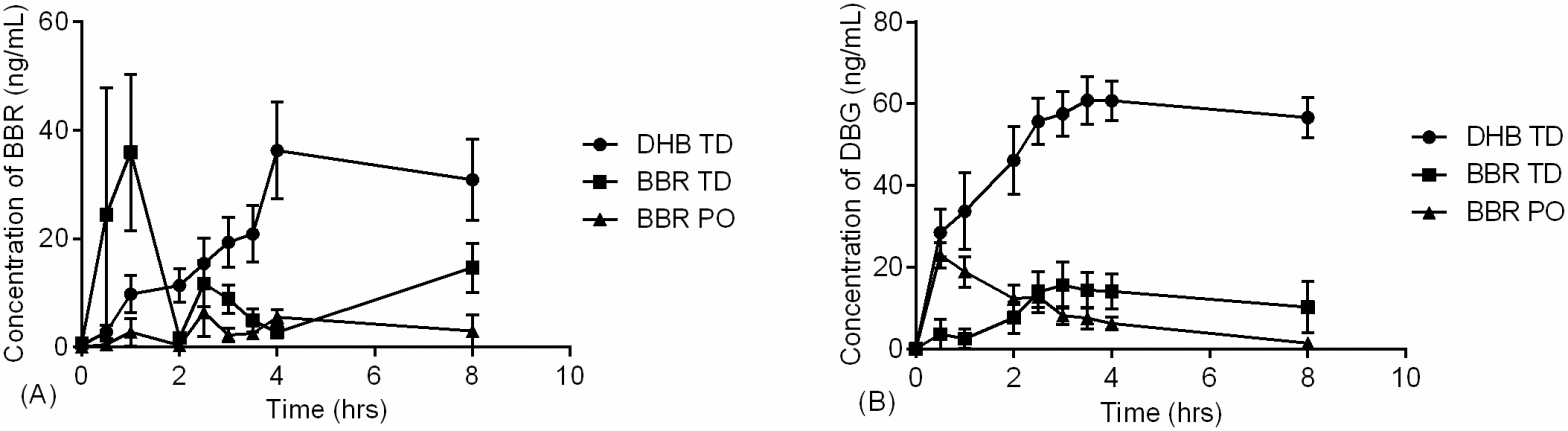
Comparative acute pharmacokinetics of formulations. Male Sprague-Dawley rats received a single administration of 90 mg/kg of active via BBR oral gavage (BBR PO; ▲; N = 3), 5% (w/w) BBR transdermal formulation (BBR TD; -■-; N = 8), or 5% (w/w) DHB transdermal formulation (DHB TD; -●-; N = 8) and serum was collected over the course of eight hours. Error bars represent standard error of the mean.

The pharmacokinetics of berberine after acute oral administration in rats was comparable to that found in previous reports [16] and devoid of any observable acute adverse events. Berberine blood levels resulting from topical administration has never been investigated to our knowledge and displayed enhanced bioavailability (3.6 x AUC_0-8_, p < 0.05) upon acute administration compared with orally treated animals (Table 5). While BBR TD yielded higher berberine bioavailability (AUC_0-8_; 95.6 +/- 23.7 ng·h/mL) as compared to oral (26.5 +/- 2.9 ng·h/mL) this difference between routes of administration was not reflected in metabolite levels (Table 5). We hypothesize these results reflect the distinction between routes in terms of first-pass hepatic metabolism, wherein transdermal compounds bypass the liver while entering the systemic circulation and may also have distinct tissue distributions. Turner et. al., reported that oral DHB was more bioavailable then oral berberine [40], an observation that was reinforced by Feng et. al. [28], who added that DHB converts to BBR rapidly in solution and in blood, and therefore berberine is an appropriate analyte for either dosage form. We found that the enhanced oral bioavailability of dihydroberberine translates to improved transdermal permeation over both oral berberine (7.1 x AUC_0-8_, p <0.05) and topical berberine (2.0 x AUC_0-8_) after a single dose. This may be related to the increased lipophilicity of DHB compared with BBR. The time to maximum serum concentration (t_max_) of berberine varied noticeably from group to group, occurring the earliest for BBR TD (Fig 3A). The maximum berberine serum concentrations occurred at 4h for DHB TD and yielded an overall AUC_0-8_ of 187.3 ng·h/mL, significantly higher than oral BBR (Table 5). This suggests that DHB TD can yield ~7-fold higher berberine bioavailability compared to oral BBR. An additional DHB oral group would have been beneficial to compare the utility of TD DHB, unfortunately due to the challenges in solubility and stability of DHB further development is required to ensure it is administered in its intended form.

**Table 5:** Comparative acute pharmacokinetic parameters of BBR treatment groups.

Analyte	Treatment group	C_max_ (ng/mL) (mean +/- SEM)	T_max_ (h)	AUC (ng·h/mL) (mean +/- SEM)
BBR	DHB TD	36.3 +/- 9.0	4	187.3* +/- 35.0
	BBR TD	36.0 +/- 14.4	1	95.6* +/- 23.7
	BBR PO	6.4 +/- 4.5	2.5	26.5 +/- 2.9
DBG	DHB TD	65.0^†^ +/- 4.6	3.5	411.1*^†^ +/- 29.3
	BBR TD	15.7 +/- 5.7	3	83.5 +/- 34.8
	BBR PO	22.9 +/- 3.1	0.5	66.0 +/- 2.1

*Statistical significance as compared to BBR PO (*P<0.05)) or compared to BBR TD (^†^ P<0.05) were determined by one-way ANOVA.

Although topical applications (BBR TD or DHB TD) produce measurable berberine levels in circulation, it is unclear if this results in accumulation of berberine within target tissues, such as the liver [41]. To address this and gain insight into berberine metabolism we tandemly quantified a previously identified metabolite, demethylene berberine glucuronide (DBG) [31]. Systemic DBG concentration (AUC_0-8_) was significantly higher for DHB TD as compared to all other treatment groups (Fig 3B & Table 5). In general DHB TD yielded a berberine and DBG pharmacokinetic pattern approaching zero-order kinetics typical of transdermal applications [42].

### Chronic berberine pharmacokinetics and liver function

**Fig 4:**
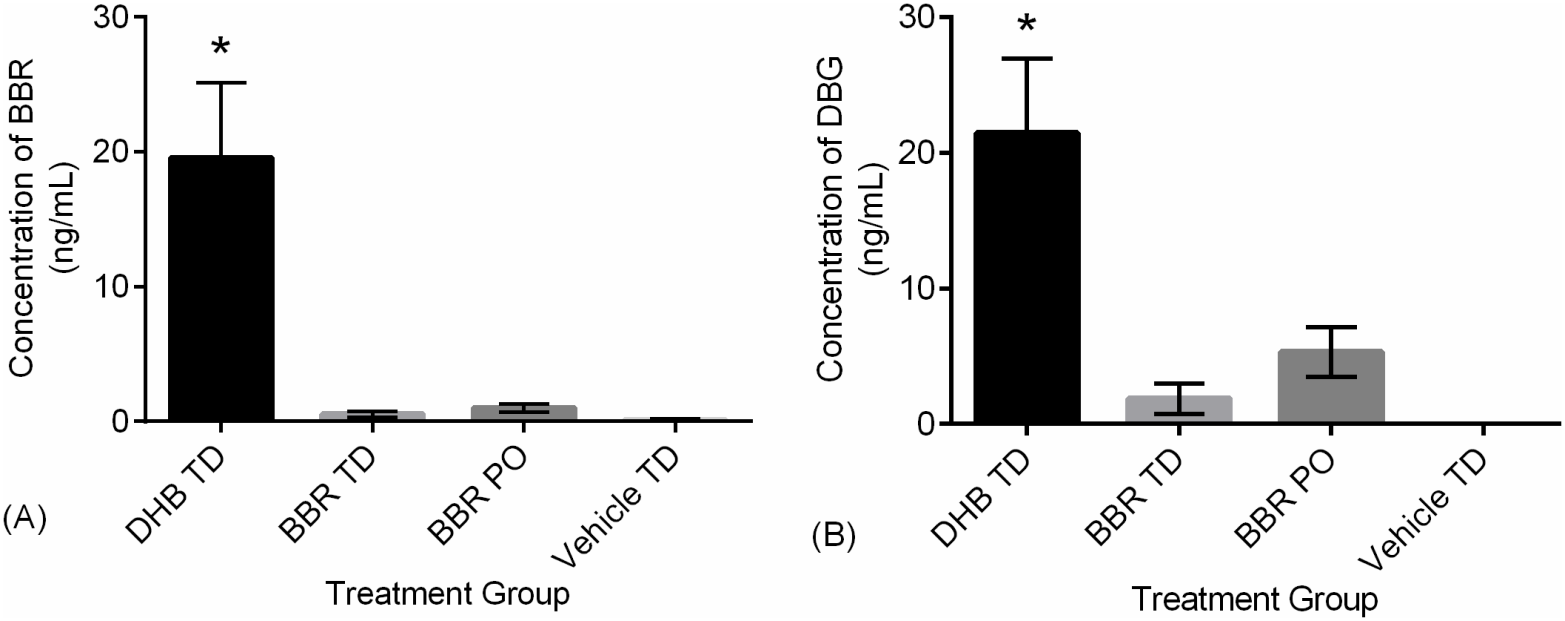
Comparative bioavailability of BBR formulations after 14 days of chronic administration. Male Sprague-Dawley rats (N = 4/group) received once daily for 14 days administration of 90 mg/kg active via BBR oral gavage (BBR PO), 5% (w/w) BBR transdermal formulation (BBR TD), and 5% (w/w) DHB transdermal formulation (DHB TD) or vehicle control (Vehicle TD). Serum was collected on day 14, two hours post-administration. Concentrations were measured by LC-MS/MS and (A) BBR levels were 19.6, 0.5, 1.0, and 0.1 ng/mL and (B) Relative DBG levels were 21.5, 1.9, 5.3, and 0 ng/mL for DHB TD, BBR TD, BBR PO, and Vehicle TD respectively. Statistical significance as compared to all other groups (*P<0.05) is indicated. Error bars represent standard error of the mean.

While acute pharmacokinetics support the theory that transdermal administration of BBR and DHB overcome the poor bioavailability of oral administration, this natural supplement consumed by humans on a regular daily basis. Therefore, this optimized formulation and route of administration should be evaluated in both acute and chronic settings. As such, SD rats were administered these same formulations along with a vehicle TD once daily for a period of 14 days and serum was quantified for BBR and DBG (Fig 4). This duration of treatment (14 days) was chosen to aid in identifying any overt issues with chronic use and is presumed sufficient to achieve steady-state kinetics. During this experiment no statistical change in body weight or Functional Observational Battery (FOB) testing was observed as a response to chronic treatment. However, mild skin redness appeared transiently in some animals within all transdermal groups. Chronic treatment with DHB TD led to significantly higher concentrations of circulating berberine (Fig 4A) and DBG (Fig 4B). This was consistent with the higher bioavailability observed in the single-dose DHB TD animals. Worthy of note is the clear distinction between DHB TD and BBR TD, with the latter generating levels approaching the lower limits of quantification. Indeed, this model of chronic administration supports a bioavailability ranking of DHB TD >>> BBR PO > BBR TD.

**Fig 5:**
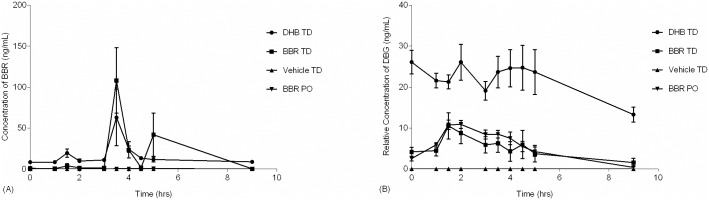
Comparative pharmacokinetics of formulations after 16 days of administration. Male Sprague-Dawley rats (N = 4/group) received once daily for 16 days administration of 90 mg/kg active via BBR oral gavage (BBR PO; ▼), 5% (w/w) BBR transdermal formulation (BBR TD; -■-), 5% (w/w) DHB transdermal formulation (DHB TD; -●-), or vehicle control (Vehicle TD; ▲) and serum collected over the course of nine hours. Error bars represent standard error of the mean.

To appropriately describe berberine pharmacokinetics during chronic administration, a complete pharmacokinetic assessment was completed following 16 days of once daily treatment (Fig 5). DHB TD demonstrated a significantly higher berberine AUC_0-9_ bioavailability as compared to oral (Fig 5A & Table 6). In addition, the DHB TD had comparably higher AUC_0-9_ for DBG than all other groups including the BBR TD. Of note, the serum levels of berberine at time-zero (Fig 5) are comparable to those described in a separate cohort (Fig 4), which illustrates DHB TD as the most bioavailable formulation. Moreover, the levels of berberine and the metabolite DBG in circulation at time-zero are similar to those at the last observed time point, which suggests the dose and once daily regimen of DHB TD may yield a steady-state presence of berberine.

**Table 6:** Comparative chronic pharmacokinetic parameters of treatment groups.

Analyte	Treatment group	C_max_ (ng/mL) (mean +/- SEM)	T_max_ (h)	AUC (ng·h/mL) (mean +/- SEM)
BBR	DHB TD	57.1* +/- 91.7	3.5	149.7*+/- 10.4
	BBR TD	108.1* +/- 54.0	3.5	142.5* +/- 99.8
	BBR PO	1.1 +/- 0.6	0	4.8 +/- 0.9
	Vehicle TD	0.0 +/- 0.0	N/A	0.0 +/- 0.0
DBG	DHB TD	26.1 +/- 6.4	4	190.7 +/- 43.0
	BBR TD	10.5 +/- 4.8	1.5	41.4 +/- 21.6
	BBR PO	10.9 +/- 1.2	2	47.7 +/- 4.6
	Vehicle TD	0.0 +/- 0.0	N/A	0.0 +/- 0.0

*Statistical significance as compared to BBR PO (*P<0.05) was determined by one-way ANOVA.

Comparing pharmacokinetic parameters between acute and chronic experiments, there was no statistically significant change in berberine AUC levels for BBR TD (95.6 +/-23.7 vs. 142.5 +/-99.8) or DHB TD (187.3 +/-35.0 vs. 149.7 +/-10.4). In contrast, chronic oral administration demonstrates a ~5-fold decrease (26.5 +/-2.9 vs. 4.8 +/-0.9; P = 0.0004, unpaired t-test) for berberine, possibly indicating a change in bioavailability which may be related to changes in the gastro-intestinal tract. Comparing pharmacokinetic parameters for DBG between acute and chronic experiments, the distinction between routes of administration is not present. For both BBR PO and DHB TD there is an apparent decrease in DBG AUC values from acute to chronic usage (66.0 +/-2.1 vs 47.7 +/-4.6 and 411.1 +/-29.3 vs. 190.7 +/-43.0, respectively). This decrease in DBG may reflect an alteration in hepatic function such as changes in enzymes responsible for berberine metabolism, of which CYP3A4 is a significant player [43–45].

**Fig 6:**
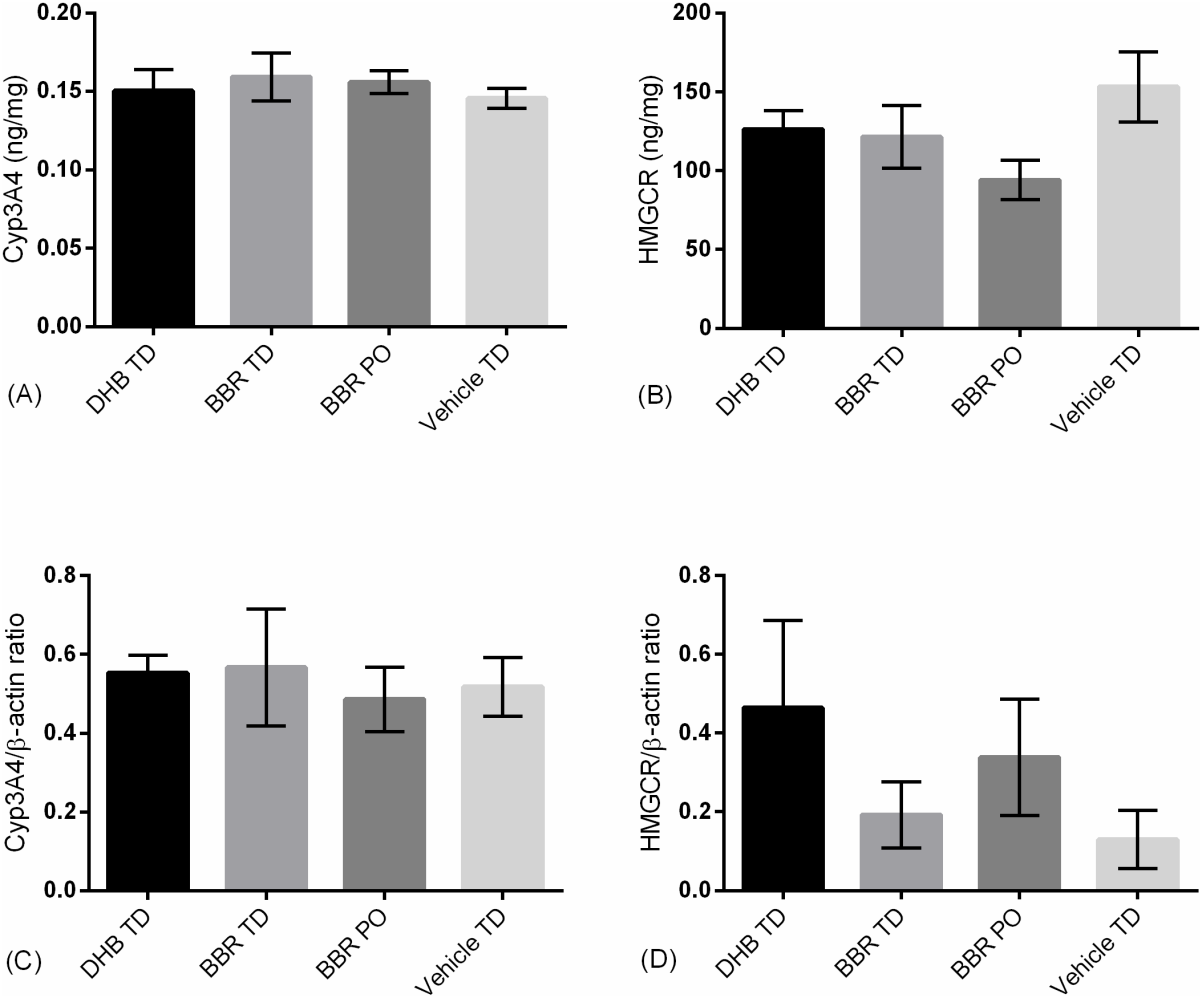
Liver expression levels of Cyp3A4 and HMG-CoA in chronically administered animals. Male Sprague-Dawley rats (N = 4/group) received once daily for 14 days administration of 90 mg/kg active via BBR oral gavage (BBR PO), 5% (w/w) BBR transdermal formulation (BBR TD), and 5% (w/w) DHB transdermal formulation (DHB TD) or vehicle control (Vehicle TD). Two liver samples/group were lysed and evaluated using commercially available colorimetric ELISAs for (A) CYP3A4 and (B) HMG-CoA reductase or using Western blot densitometry bioassays with β-actin as reference signal for (C) CYP3A4 and (D) HMG-CoA Reductase. No statistical difference between groups was observed (Example western blots provided in S1 Fig). Error bars represent standard error of the mean.

To address this possibility, liver samples from chronically treated animals (14 days) were dissected and CYP3A4 was quantified by ELISA and western blot (Fig 6). No differences in CYP3A4 expression were observed between treatment groups. In addition, Wu et. Al. reported chronic oral berberine does not affect hepatic HMG-CoA reductase expression [46], an observation replicated in this study for oral and transdermal formulations. Together, 14 day chronic administration and the enhanced bioavailability of DHB TD yields no overt alteration in hepatic xenobiotic metabolism (Fig 6A) nor alters key rate-limiting enzymes (Fig 6B) in this preliminary investigation.

**Fig 7:**
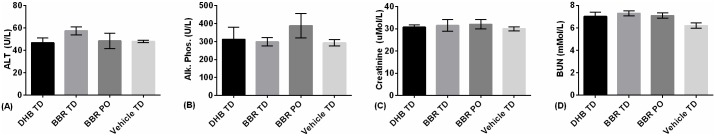
Clinical chemistry of liver and kidney function in chronically administered animals. Male Sprague-Dawley rats (N = 4/group) received once daily for 14 days administration of 90 mg/kg active via BBR oral gavage (BBR PO), 5% (w/w) BBR transdermal formulation (BBR TD), and 5% (w/w) DHB transdermal formulation (DHB TD) or vehicle control (Vehicle TD). Whole blood was collected on day 14 and analyzed for biomarkers of hepatic and nephrotic function including (A) ALT, (B) ALP, (C) creatinine, and (D) BUN. Error bars represent standard error of the mean.

Furthermore, the absence of overt organ disruption is confirmed for both liver and kidneys. Blood clinical chemistry was performed at day 14 and included the evaluation of alanine aminotransferase (ALT), alkaline phosphatase (ALP), creatinine, and blood urea nitrogen (BUN) (Fig 7). Analysis of variance of clinical markers yielded no statistically significant differences between groups, and the overall levels are not diagnostic of a deleterious event when compared to acceptable veterinary ranges [47,48].

Simvastatin and berberine both undergo metabolism in the liver primarily through CYP3A4 [44,49]. Furthermore, Liu et. al. [36], found that simvastatin treatment significantly increased plasma concentrations of berberine in animals that were administered a single dose of each, vs. animals that were treated with berberine alone. As such, we set about evaluating the interaction between chronic berberine administration and acute simvastatin treatment.

**Fig 8:**
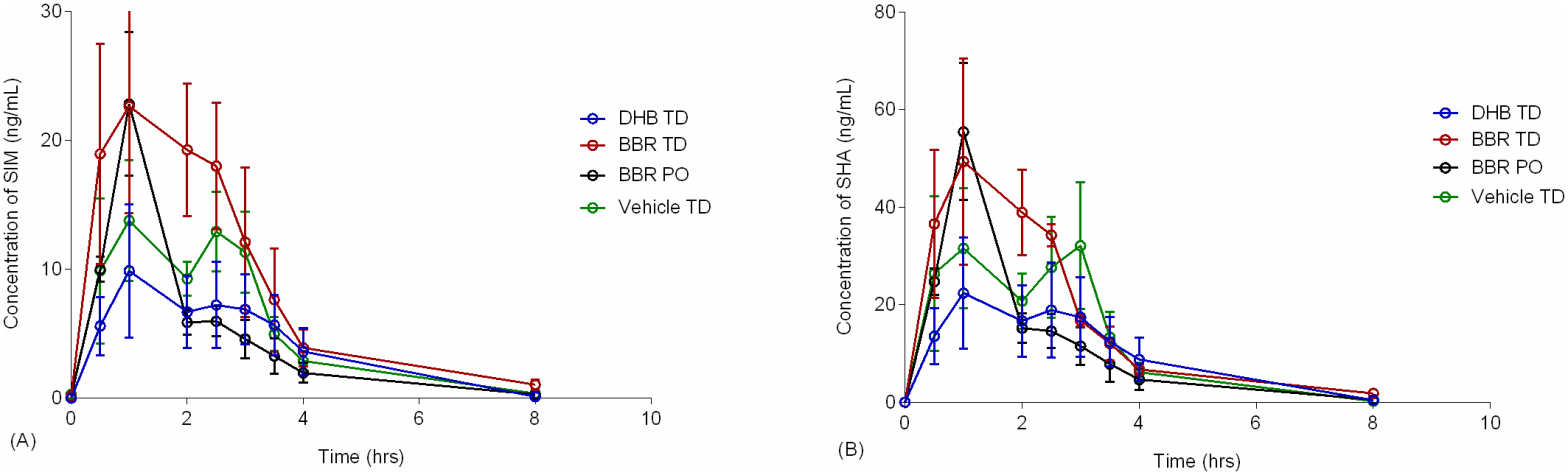
Comparative pharmacokinetics of SIM and SHA after 16 days of BBR treatment. Male Sprague-Dawley rats (N = 4/group) received once daily for 16 days administration of 90 mg/kg of active via BBR oral gavage (BBR PO, designated with green ο), 5% (w/w) BBR transdermal formulation (BBR TD, designated with red ο), 5% (w/w) DHB transdermal formulation (DHB TD; designated with blue ο), or vehicle control (vehicle-TD, designated with black ο). On day 16 all animals also received 12 mg/kg simvastatin and serum was collected over the course of eight hours. Concentrations of (A) SIM and (B) SHA were quantified. No statistical difference between groups was observed (S2 Table). Error bars represent standard error of the mean.

Pharmacokinetics of simvastatin was compared between SD rats treated with an active and control (Fig 8, S2 Table). There was no significant difference in AUC_0-8_ or C_max_ values for either SHA or SIM between groups treated with the active and the control groups or between positive treatment groups. This is consistent with a finding by Liu et all that a single, co-administered dose of oral berberine did not influence single dose simvastatin pharmacokinetics [36]. The shape of the drug concentration-time curves did vary slightly between groups (Fig 8), however there was no statistical difference in AUC_0-9_ between treatment (oral or transdermal) and the vehicle control (S2 Table) Based upon these results there is no cumulative change in simvastatin bioavailability in animals treated chronically with berberine formulations, suggesting co-administration of these two actives for therapeutic value could be considered However, the results discussed here are preliminary and additional testing is required to investigate this important issue.

## Conclusion

Multi-analyte quantification in low volumes of rat serum was facilitated by the development of a concurrent and sensitive LC-MS/MS method. This analytical method was applied to pharmacokinetic experiments comparing oral berberine, transdermal berberine, and transdermal dihydroberberine in both acute and chronic studies. Transdermal dihydroberberine presented increased systemic berberine and DBG compared with other routes of administration in both acute and chronic treatment. Interestingly, a decrease in bioavailability between acute and chronic experiments suggests changes in gastrointestinal handling and hepatic metabolism, with the former overcome through transdermal administration. While transdermal dihydroberberine unequivocally increased berberine levels in circulation this increase had no effect on liver or kidney function nor altered the expression of hepatic enzymes CYP3A4 and HMG-CoA reductase. Furthermore, chronic administration of berberine or dihydroberberine yielded no overt change in simvastatin bioavailability suggesting these two actives may investigated in tandem. While it remains to be determined if transdermal dihydroberberine could yield improved bioavailability compared its oral equivalent, the opportunity that topical routing presents with avoidance of the gastrointestinal tract and first-pass metabolism is worthy of continued investigation. In summary, the introduction of berberine by means of its precursor dihydroberberine within the transdermal formulation may prove significantly advantageous and supports further research into this formulations efficacy in models of diabetes or dyslipidemia.

## Supporting information

S1 Fig Liver expression levels of Cyp3A4 and HMG-CoA in chronically administered animals using western blot. Male Sprague-Dawley rats (N = 4/group) received once daily for 14 days administration of 90 mg/kg active via BBR oral gavage (BBR PO), 5% (w/w) BBR transdermal formulation (BBR TD), and 5% (w/w) DHB transdermal formulation (DHB TD) or vehicle control (Vehicle TD). Examples of westn blot data used for densitometric semi-quantification are present for (A) CYP3A4 and (B) its corresponding β-actin and (C) HMG-CoA reductase and (D) its corresponding β-actin.
(PDF)

S1 Table Stability of berberine, simvastatin and simvastatin hydroxy acid. Six replicates of QC samples (spiked serum) were analyzed for each analyte at each concentration after being subjected to the following sets of conditions: three freeze/thaw cycles at—20 °C, long term freezing storage (4 weeks at—80 °C), and 3 hours at room temperature. Stock solutions and samples prepared in serum extracts were also monitored for stability for up to 5 days at room temperature.
(PDF)

S2 Table Pharmacokinetic parameters of simvastatin and simvastatin hydroxy acid after oral administration of 12 mg/kg simvastatin. Each Value Represents Mean +/- SEM. Statistical analysis was completed using one-way ANOVA, without detection of significance.
(PDF)
