# Supplementary material for: Comparative pharmacokinetics and safety assessment of transdermal berberine and dihydroberberine
Source: PLoS One. 2018 Mar 26;13(3):e0194979. doi: 10.1371/journal.pone.0194979 (PMC5868852; doi:10.1371/journal.pone.0194979)

**S1 Fig. Liver expression levels of Cyp3A4 and HMG-CoA in chronically administered animals.**

| | | | | | | | | |
|---|---|---|---|---|---|---|---|---|
| CTRL | CTRL | BBR PO | BBR PO | BBR TD | BBR TD | DHB TD | DHB TD | Vehicle TD |

CYP3A4

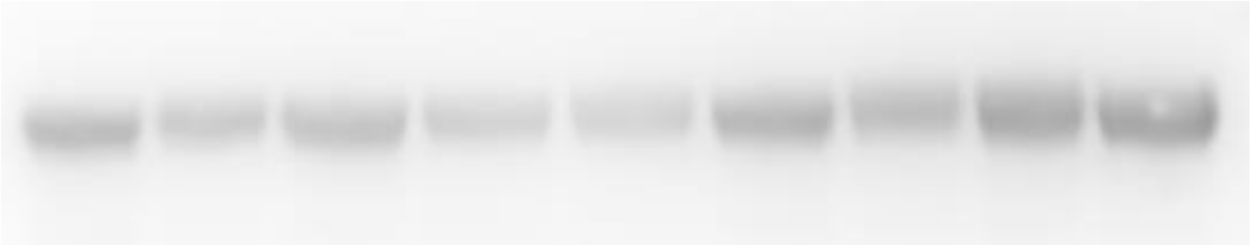

B-Actin

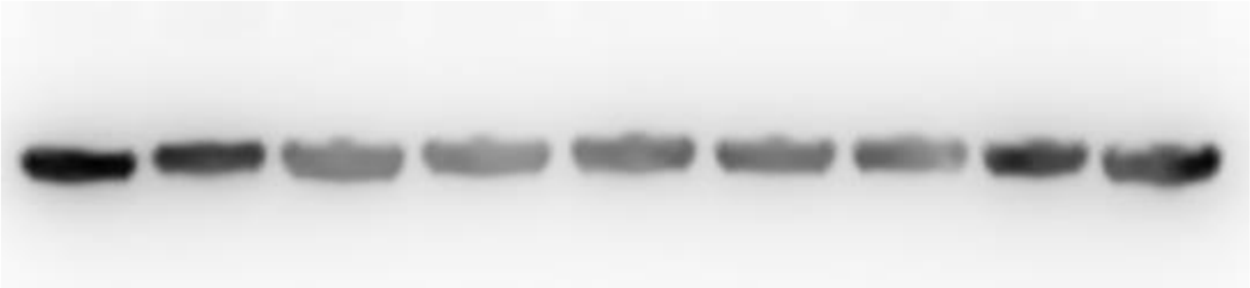

HMG CoA

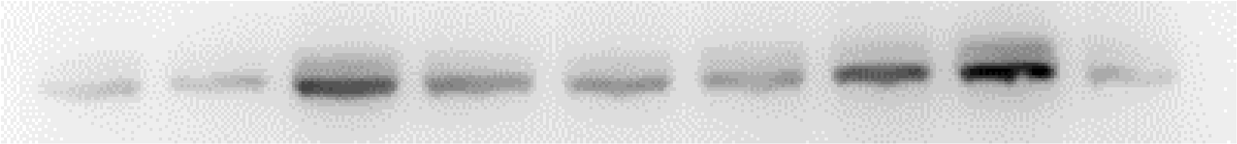

B-Actin

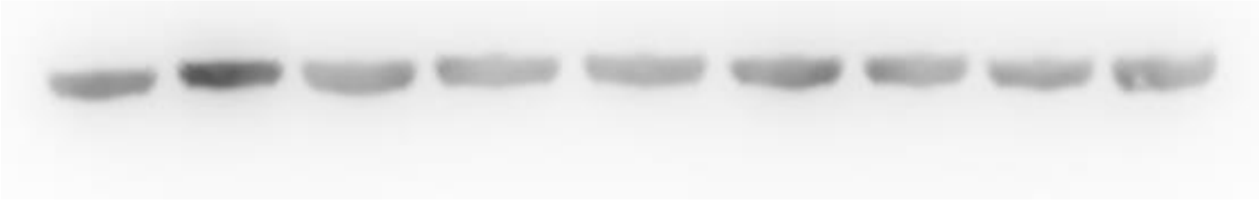

Supplement: S1 Fig — Male Sprague-Dawley rats (N = 4/group) received once daily for 14 days administration of 90 mg/kg active via BBR oral gavage (BBR PO), 5% (w/w) BBR transdermal formulation (BBR TD), and 5% (w/w) DHB transdermal formulation (DHB TD) or vehicle control (Vehicle TD). Examples of westn blot data used for densitometric semi-quantification are present for (A) CYP3A4 and (B) its corresponding β-actin and (C) HMG-CoA reductase and (D) its corresponding β-actin. (PDF) [file pone.0194979.s001.pdf]
