# Supplementary material for: Comparative pharmacokinetics and safety assessment of transdermal berberine and dihydroberberine
Source: PLoS One. 2018 Mar 26;13(3):e0194979. doi: 10.1371/journal.pone.0194979 (PMC5868852; doi:10.1371/journal.pone.0194979)
Supplement: S1 Table — Six replicates of QC samples (spiked serum) were analyzed for each analyte at each concentration after being subjected to the following sets of conditions: three freeze/thaw cycles at—20 °C, long term freezing storage (4 weeks at—80 °C), and 3 hours at room temperature. Stock solutions and samples prepared in serum extracts were also monitored for stability for up to 5 days at room temperature. (PDF) [file pone.0194979.s002.pdf]

S1 Table. Stability of Berberine, Simvastatin and Simvastatin Hydroxy Acid

**Storage Condition**	**Nominal Concentration (ng/mL)**	**% of Initial Concentration Remaining (% +/- SEM)**		
		Berberine	Simvastatin	Simvastatin hydroxy acid
Room temperature (spiked serum)	1.4	104.3 +/- 5.6	61.6 +/- 4.3	103.8 +/- 5.6
	5	99.6 +/- 3.1	74.2 +/- 1.6	108.0 +/- 3.9
	25	100.8 +/- 0.7	72.9 +/- 0.5	106.6 +/- 4.4
Long-term stability (- 20 ^{o}C - spiked serum)	1.4	97.3 +/- 2.5	52.3 +/- 1.2	86.3+/- 4.0
	5	91.7 +/- 5.5	42.1 +/- 1.3	85.0+/- 5.9
	25	91.8 +/- 1.0	46.0 +/- 1.9	87.4 +/- 3.6
Freeze-thaw stability (spiked serum)	1.4	96.1 +/- 0.9	26.6 +/- 3.4	101.9 +/- 4.5
	5	92.6 +/- 3.3	41.2 +/- 0.4	97.0 +/- 2.6
	25	96.1 +/- 2.2	38.7 +/- 0.7	89.2 +/- 3.9
Room temperature (spiked serum extract)	1.4	95.5 +/- 1.9	99.7 +/- 3.2	96.4 +/- 1.4
	5	98.9 +/- 0.5	97.9 +/- 2.3	98.8 +/- 0.3
	25	98.4 +/- 1.5	101.6 +/- 1.9	99.1 +/- 1.4
Room temperature (stock solution)	1.4	98.9 +/- 1.1	112.5 +/- 3.7	101.0 +/- 2.1
	5	101.4 +/- 1.7	105.1 +/- 1.4	98.6 +/- 1.1
	25	101.2 +/- 1.6	98.8 +/- 0.6	100.7 +/- 0.7
