# Supplementary material for: Comparative pharmacokinetics and safety assessment of transdermal berberine and dihydroberberine
Source: PLoS One. 2018 Mar 26;13(3):e0194979. doi: 10.1371/journal.pone.0194979 (PMC5868852; doi:10.1371/journal.pone.0194979)
Supplement: S2 Table — Each Value Represents Mean +/- SEM. Statistical analysis was completed using one-way ANOVA, without detection of significance. (PDF) [file pone.0194979.s003.pdf]

S2 Table. Pharmacokinetic Parameters of Simvastatin and Simvastatin Hydroxy Acid after Oral Administration of 12 mg/kg Simvastatin. Each Value Represents Mean +/- SEM.

**Analyte**	**Treatment Group**	**T_{max} (h)**	**C_{max} (ng/mL) (mean +/- SEM)**	**AUC_{0-8} (ng·h/mL) (mean +/- SEM)**	**$T_{1/2}$ (h) (mean +/- SEM)**
Simvastatin	DHB TD	1	9.9 +/- 5.2	32.9 +/- 10.7	1.03 +/- 0.40
	BBR TD	1	22.7 +/- 8.3	70.8 +/- 17.5	1.46 +/- 0.26
	BBR PO	1	22.8 +/- 5.8	38.6 +/- 3.5	1.18 +/- 0.35
	Vehicle TD	1	13.8 +/- 4.7	44.0 +/- 4.9	1.07 +/- 0.21
Simvastatin hydroxy acid	DHB TD	1	22.4 +/- 11.4	78.9 +/- 28.4	1.12 +/- 0.30
	BBR TD	1	49.4 +/- 21.2	136.4 +/- 25.1	1.42 +/- 0.27
	BBR PO	1	55.4 +/- 14.0	93.9 +/- 9.3	1.23 +/- 0.17
	Vehicle TD	3	32.1 +/- 13.0	104.3 +/- 24.5	0.83 +/- 0.14
